# Metagenomic sequencing of a Patescibacteria-containing enrichment from Zodletone spring in Oklahoma, USA

**DOI:** 10.1128/mra.00114-24

**Published:** 2024-03-18

**Authors:** Sayali A. Mulay, C. Ryan Hahn, Dawn M. Klingeman, Mostafa S. Elshahed, Noha H. Youssef, Mircea Podar

**Affiliations:** 1 Department of Microbiology, University of Tennessee Knoxville, Knoxville, Tennessee, USA; 2 Department of Microbiology and Molecular Genetics, Oklahoma State University, Stillwater, Oklahoma, USA; 3 Biosciences Division, Oak Ridge National Laboratory, Oak Ridge, Tennessee, USA; University of Southern California, Los Angeles, California, USA

**Keywords:** metagenome, Patescibacteria, enrichment

## Abstract

An enrichment of sulfidic sediments from Zodletone spring was sequenced as a metagenome. Draft genomes representing Cloacimonadota, Deltabacterota, Firmicutes, and Patescibacteria were binned and annotated and will aid functional genomics and cultivation efforts.

## ANNOUNCEMENT

Zodletone spring is a sulfidic spring in Kiowa County, Oklahoma (GPS coordinates 35.002444 N, 98.688167 W), used as a model environment to study microbial physiological adaptations to redox gradients resembling the great oxygenation event of early Earth (1). Among the over 60 archaeal and bacterial high-level taxa that inhabit Zodletone, Patescibacteria are highly represented and include more than two dozen largely uncultured lineages originally assigned under the bacterial “Candidate Phyla Radiation” (2–4). Zodletone sediment samples (50 mL) were collected as described (2) and were maintained anoxic and refrigerated in glass bottles with nitrogen headspace. Enrichments were generated by inoculating ~1 mL sediment into 10 mL of Pfennig’s mineral medium containing 0.12% yeast extract, 8 mM sodium sulfite, reduced with 5 mM cysteine under 85% N_2_, 10% CO_2_ and 5% H_2_, followed by incubation at 25°C and weekly passaged (1:10) in fresh medium. We obtained a Zodletone sediment anaerobic enrichment dominated by Deltabacterota [>50%, based on SSU rRNA amplicons, performed as described in reference (2)], including a *Desulfomicrobium* we have already isolated in pure culture and sequenced to completion (5) as well as *Desulfovibrio, Desulfuromonas,* and *Desulfocapsa*. The enrichment contained Patescibacteria, represented by Absconditabacterales (formerly SR1, candidate class Gracilibacteria) and Moranbacterales (candidate class Paceibacteria) at relatively low levels (<5%), as well as Spirochaetota (*Sphaerochaeta*), Cloacimonadota, and Firmicutes.

For metagenomic DNA isolation, the enrichment was grown for 7 days at 25°C in 10 mL medium. All experimental protocols followed manufacturers’ instructions. DNA was isolated using the ZymoBIOMICS DNA Miniprep Kit (Zymo Research, Irvine, CA). A short insert library was prepared using the Illumina Nextera XT DNA Library Preparation Kit. Sequencing (Illumina MiSeq Reagent Kit v2, 2 × 250-nucleotide reads) on a MiSeq instrument (Illumina, Inc., San Diego, CA), yielded 11.6 million paired reads (236 nt average length). The sequences were imported in KBase (6) and analyzed following the KBase standard metagenomic workflow (7) using default settings unless otherwise noted. Trimmomatic v0.36 (8) was used for quality-based trimming and to remove reads shorter than 150 nt. The reads were assembled using metaSPAdes v3.15.3 (9), MEGAHIT v1.2.9 (10), and IDBA-UD v1.1.3 (11) and the assemblies statistics compared with QUAST v4.4 (12). The metaSPADES assembly was selected for binning based on highest scores. The best output in terms of genome completion and contamination score, as assessed by CheckM v1.0.18 (13), was generated by MetaBAT2 v1.7 (14), which generated 21 bins (metagenome-assembled genomes, MAGs). The MAGs were taxonomically classified with GTDB-Tk v2.3.2 (15). The most complete (85%–97%) and least contaminated (<2.5%), based on single copy gene statistics, belong to Desulfobacterota (*Desulfolutivibrio, Pseudodesulfovibrio, Desulfovibrio*), Cloacimonadota, and Acholeplasmatales. Less complete MAGs (<70%) represented *Sphaerochaeta, Desulfocapsa, Desulfomicrobium,* and four MAGs of Bacillota. Our primary target group, Patescibacteria, included MAGs for an Absconditabacterales and a Moranbacterales (65%–85% complete, <1% contamination). For microbes with reduced genomes such as Patescibacteria (16), completion based on universal single copy genes is underestimated. For example, the closed genome of *Cand*. Absconditicoccus praedator, an Absconditabacterales (17), is predicted as 80% complete by CheckM.

To predict and annotate the genes in the high-quality MAGs, we used the NCBI Prokaryotic Genome Annotation Pipeline (PGAP) v6.1 (18). Table 1 lists the genome-based classification and MAGs/gene content information. The genomes and their functional annotations will facilitate mass-spectrometry proteomics and metabolic predictions to aid cultivation of individual bacteria from that enrichment using selective media and targeted reverse genomics (19).

**TABLE 1:** Accession numbers and characteristics of metagenome-assembled genomes from Zodletone spring enrichment^*a*^

MAG	Size (Mbp)	Comp (%)	Cont (%)	Classification	G + C %	Genbank	Contigs	N50 (kb)	Proteins	RNAs
B1-Zod	3.8	97	0.8	Desulfobacterota_Desulfolutivibrio	63.6	JAZHDB000000000	238	26.2	3500	53
B6-Zod	1.6	66^*b*^	0.9	Patescibacteria_Absconditabacterales	26.8	JAZHDC000000000	198	9.3	1397	31
B10-Zod	3.1	91	0	Desulfobacterota_Desulfovibrionaceae	59.4	JAZHDD000000000	71	67.7	2891	38
B12-Zod	2.2	86	0.5	Cloacimonadota_PHBP01	49.3	JAZHEB000000000	279	8.6	1823	43
B13-Zod	1.2	85^*b*^	0.7	Patescibacteria_Moranbacterales	37.5	JAZHDF000000000	97	16.6	1117	41
B16-Zod	1.2	91	0.9	Bacillota_Acholeplasmatales UBA6181	33.5	JAZHDG000000000	105	17.4	1176	30
B21-Zod	3.3	88	2.5	Desulfobacterota_Pseudodesulfovibrio	64.6	JAZHDH000000000	326	15.7	3180	39

^*a*^ Comp, completeness; Cont, contamination.

^*b*^ Likely underestimated due to streamlining.

## Data Availability

The metagenome sequence reads are available in the NCBI Sequence Read Archive (SRA) under the accession number SRR27793275. The annotated MAGs have been deposited in GenBank under the accession numbers JAZHDB000000000, JAZHDC000000000 JAZHDD000000000, JAZHDE000000000, JAZHDF000000000, JAZHDG000000000, and JAZHDH000000000.
